# miR-26a and miR-214 down-regulate expression of the PTEN gene in chronic lymphocytic leukemia, but not PTEN mutation or promoter methylation

**DOI:** 10.18632/oncotarget.2626

**Published:** 2014-10-24

**Authors:** Zhi-Jian Zou, Lei Fan, Li Wang, Ji Xu, Run Zhang, Tian Tian, Jian-Yong Li, Wei Xu

**Affiliations:** ^1^ Department of Hematology, the First Affiliated Hospital of Nanjing Medical University, Jiangsu Province Hospital, Nanjing, China; ^2^ Department of Hematology, Wuxi People Hospital Affiliated of Nanjing Medical University, Wuxi, China

**Keywords:** chronic lymphocytic leukemia, PTEN, mutation, microRNA

## Abstract

We previous found the expression level of PTEN was low in the chronic lymphocytic leukemia (CLL) patients. To assess the pathogenic contribution of the low expression of PTEN, we determined PTEN-regulating miRNA interference, PTEN promoter methylation and PTEN gene mutation condition in CLL.

One hundred and fifty-four previously untreated CLL patients and 200 cases of healthy controls were sequenced in exons 5−9 of PTEN. None of single nucleotide polymorphism site or mutation was detected in the coding sequences of those exons. Methylation of PTEN promoter was found in one (1.33%) of the 75 patients with CLL, but none of the 25 age-matched control subjects. We found that PTEN was a potential target of miR-26a and miR-214, which had been confirmed following dual-luciferase reporter assays, reverse transcription polymerase chain reaction and Western blotting. High expression of miR-26a was associated with advanced Binet stage (P=0.012), p53 aberrations (P=0.014) and inferior time to first treatment (P=0.038), and high expression of miR-214 was only associated with p53 aberrations (P=0.041). Inhibition of miR-26a or miR-214 could induce more apoptosis in primary cultured CLL cells. These findings support miR-26a and miR-214 down-regulate expression of PTEN in CLL, but not PTEN mutation or promoter methylation.

## INTRODUCTION

Chronic lymphocytic leukemia (CLL) is the most common type of adult leukemia in the Western societies [1], but low incidence in Asian countries, including China [2, 3]. With the coming of aging society and the westernization of lifestyle, increasing incidence has been reported in recent years in Asia [4]. Although approximately one third of patients are asymptomatic and are observed without treatment at the time of diagnosis, CLL is a progressive disease that in most patients will eventually require treatment. Common treatments include alkylating agent, purine analogs and immunotherapeutic agents. However, no current treatment is curative, and all patients eventually relapse [5]. It is urgent to explore the new treatment modality. Several small molecule inhibitors which target kinases in the B cell receptor (BCR) pathway have promising clinical activity, pave the way for a revolution in the treatment of CLL [6].

Phosphatase and tension homolog deleted on chromosome ten (PTEN) acts as a tumor suppressor gene through inhibition of PI3K/AKT, which regulates cellular growth, metabolism and survival. Recent studies have demonstrated the inactivation of PTEN in lung cancer [7], breast cancer [8], glioblastomas [9], endometrial carcinoma [10,11], colorectal carcinoma [11] and hematologic malignancies [12,13]. In our previous study, we found PTEN expression was down-regulated in CLL patients compared to purified B cells from the normal controls, and the low expression level of PTEN was associated with adverse clinical prognosis [14]. Therefore, we further investigated the common mechanisms of aberrant expression of the PTEN gene in CLL, including PTEN gene mutations, promoter methylation and microRNA (miRNA) intervention.

## RESULTS

### miR-21, miR-26a and miR-214 directly targeted PTEN

**Figure 1:**
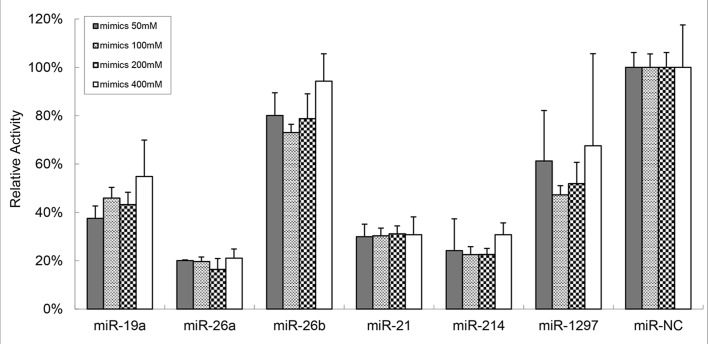
miR-21, miR-26a and miR-214 apparently targeted PTEN The dual-luciferase assay reveals that 50 nM, 100 nM, 200 nM and 400 nM of the miR-21, miR-26a and miR-214 mimics significantly suppress the activity of PTEN when compare to miR-NC (mean percent of luciferase activity reduction in the three miRNAs of 69.47%, 80.72% and 74.97%, respectively).

miRNAs can bind to target mRNA transcripts of protein-coding genes and negatively control their target genes. miRNAs were demonstrated to have a functional role in many cancers and sometimes are referred to as oncomirs. To study the mechanism of aberrant PTEN expression, we explored the correlation between PTEN-targeting miRNAs and PTEN expression in CLL patients. As shown in Supplemental Figure 1, the bioinformatic analysis identified six miRNAs as possible regulators of PTEN (miR-19a, miR-21, miR-26a, miR-26b, miR-214 and miR-1297). To further determine if PTEN was directly regulated by these miRNAs, we constructed luciferase reporter assays with 3′-untranslated regions (UTR) of PTEN (pEZX-PTEN-3′-UTR). The luciferase reporter was introduced 50 nM, 100 nM, 200 nM and 400 nM of these miRNAs mimics and miRNA negative control (miR-NC) into 293T cells. The luciferase activity was significantly suppressed in the cell line when pEZX-PTEN-3′-UTR was cotransfected with miR-21, miR-26a and miR-214 mimics (mean percent of luciferase activity reduction in the three miRNAs of 69.47%, 80.72% and 74.97% respectively, Figure 1).

### miR-26a and miR-214 inhibitors up-regulated PTEN expression and induced CLL cells apoptosis *in vitro*

**Figure 2:**
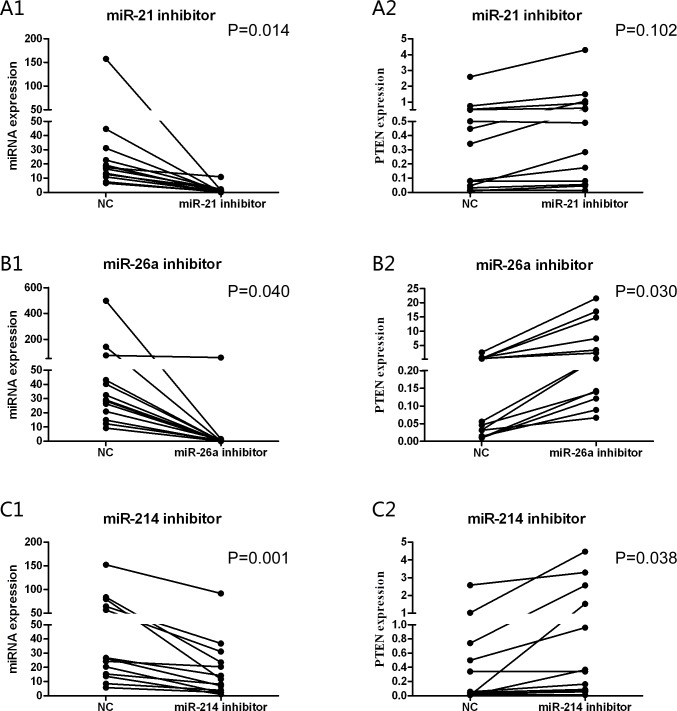
The different expression levels of miRNAs and PTEN were detected after 24 hours from transfections After antisense miRNAs transfection, the primary CLL cells express obviously increased PTEN mRNA in miR-26a (P=0.030) (B2) and miR-214 (P=0.038) (C2) inhibitors groups. While the corresponding miRNAs fall significantly in three groups compared to transfected with miR-NC (P=0.014, A1; P=0.040, B1; P=0.001, C1) separately.

Unsupervised hierarchical clustering of our earlier study showed that miR-21 and miR-26a were significantly up-regulated in CLL patients (P=0.017 and P<0.001, respectively) in comparison with in purified B cells from healthy donors [15]. Dysregulation of miRNAs may be associated with cancers. We thus hypothesized that over-expressed these miRNAs suppress PTEN expression, which inhibit cell apoptosis in the CLL patients. To investigate the anti-tumor potential of the three miRNA inhibitors, we assessed whether the down-regulation of these miRNAs affected PTEN expression in primary CLL cells. The primary cells of 13 newly diagnosed CLL cases were transiently transfected with miR-21, miR-26a, miR-214 inhibitors and miR-NC. The expression levels of miRNAs and PTEN were detected after 24 hours from transfections. We found that PTEN gene was up-regulated at mRNA level after transfection of the three miRNAs inhibitors, as compared with cells transfected with miR-NC (Figure 2,=0.102, P=0.030 and P=0.038, respectively). Simultaneously, by real-time quantitative polymerase chain reaction (RQ-PCR) analysis of CLL cells treated with these miRNAs inhibitors, we found remarkable knocking down of miR-21, miR-26a and miR-214 expression (Figure 2,=0.014, P=0.040 and P=0.001, respectively).

**Figure 3:**
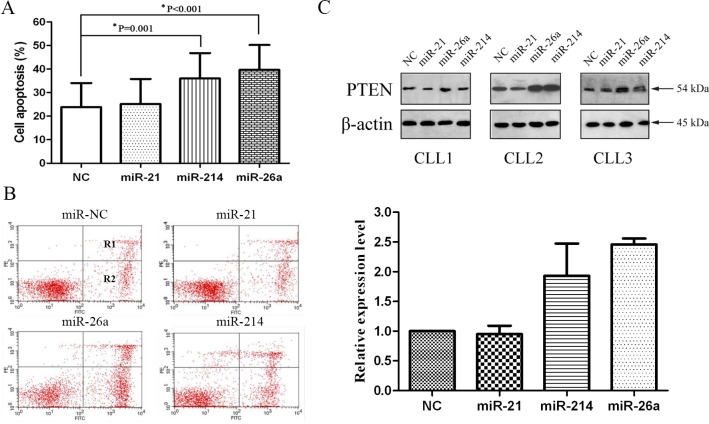
miR-26a and miR-214 inhibitors up-regulate PTEN protein expression and induce CLL cells apoptosis (A) Compared to miR-NC, miR-26a and miR-214 inhibitors groups show significantly greater average apoptosis percentages (39.90% vs. 22.88%, P<0.001; 35.61% vs. 22.88%, P=0.001). (B) Incidence of cell apoptosis in a patient of CLL. Flow cytometric analysis is shown. Y-axis: number of cells stained with PI; X-axis: cells stained with Annexin V–FITC. Apoptotic cells are in R1 and R2; Higher apoptosis percentage (R1+R2) in CLL cells treated with miR-26a (45.01% vs. 25.08%) and miR-214 (32.90% vs. 25.08%) inhibitors groups than treated with miR-NC. (C) Western blot analysis of PTEN and β-actin. Fold changes in protein levels are shown below the gel figure and are normalized to the level in miR-NC-treated cells, which is assigned a value of 1.00 (miR-26a: 2.46, miR-214: 1.93).

To investigate effects of these miRNAs inhibitors, we performed cell apoptosis assays. We found that treatment with miR-26a and miR-214 inhibitors strikingly increased the apoptosis of CLL cells (P<0.001 and P=0.001, respectively, Figure 3A and 3B). We next conducted Western blot analyses with the CLL cells treated with the three miRNAs inhibitors and miR-NC inhibitors. Western blot analysis showed that PTEN protein expression was markedly up-regulated after cell transfection with miR-26a and miR-214 inhibitors (Figure 3C). Those results showed that the up-regulation of PTEN with miR-26a and miR-214 inhibitors could enhance the apoptosis of the primary CLL cells.

### High expression of miR-26a correlates with adverse clinical characteristics and inferior time to first treatment (TTFT)

The expression levels of three potential oncomirs (miR-26a and miR-214) and PTEN mRNA were detected in 30 primary CLL cases. Interestingly, we found the expression of PTEN was associated negatively with miR-26a (r=-0.563, P=0.001, Supplemental Figure 2A) and miR-214 (r=-0.418, P=0.022, Supplemental Figure 2B). Then we analyzed correlations between the expression levels of these miRNAs and patients' clinical characteristics, including Binet stage, p53 aberrations (mutations/deletion), IGHV mutation status and TTFT. The patients were divided into cases with low and high expression based on the median expression of each miRNA in the analyzed cohort. As shown in Supplemental Figure 3, high expression of miR-26a was associated with advanced clinical Binet stage (P=0.012), p53 aberrations (P=0.014) and inferior TTFT (P=0.038), and high expression of miR-214 was only associated with p53 aberrations (P=0.041).

### PTEN gene mutation was rare in the CLL patients

**Figure 4:**
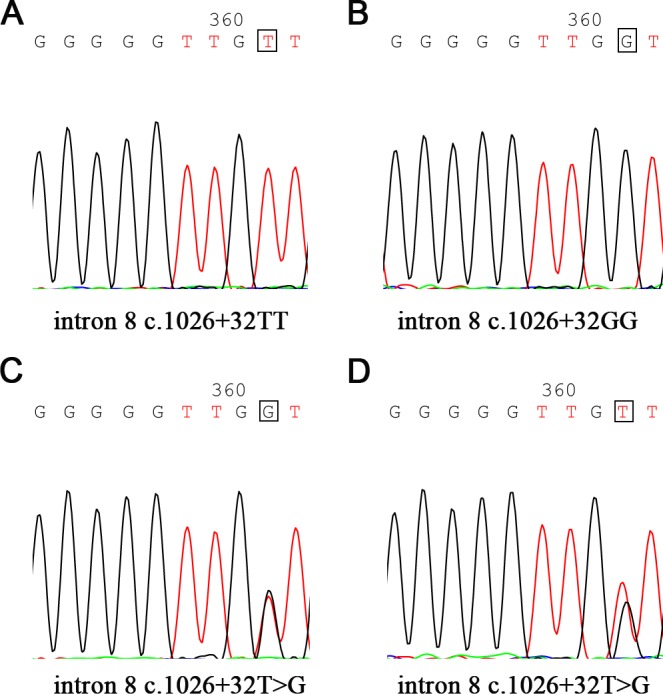
Single nucleotide polymorphism (SNP) site rs555895 in PTEN gene PTEN SNP rs555895 (intron 8 c.1026+32) genotypes are detected in 154 CLL patients and 200 healthy controls (The base in the box represents different genotype, such as T/T, G/G and T/G).

To determine the prevalence of PTEN mutations, we performed mutation analysis of PTEN exons 5−9 and flanking intronic sequences in 154 patients with newly diagnosed CLL. To our surprise, none of these CLL patients analyzed presented PTEN mutations. Nevertheless, we found patients who had intron 8 single nucleotide polymorphism (SNP) site rs555895 (intron 8 c.1026+32) (Figure 4). The rs555895 polymorphism was subsequently detected in the 200 age-matched healthy control cohorts of the Han Chinese population. Genotype distribution and allele frequencies for the SNP of PTEN in CLL patients and controls are shown in Table 1. No significant difference in distribution of the genotypes was observed between patients and controls (P=0.255). Compared to PTEN rs555895 T/T, the combined genotypes of PTEN rs555895 G/T+G/G did not show increased risk genotypes in the CLL patients (P=0.102).

**Table 1:** Genotype distribution and allele frequencies for the SNP of PTEN in CLL patients and controls

	Genotype			P	Genotype		P
	T/T	T/G	G/G		T/T	T/G+G/G	
CLL	37 (24.0%)	81 (52.6%)	36 (23.4%)	0.255	37 (24.0%)	117 (76.0%)	0.102
Control	34 (17.0%)	117 (58.5%)	49 (24.5%)		34 (17.0%)	166 (83.0%)	

### Methylation status of PTEN in CLL cohorts

PTEN was down-regulated in CLL cells, so we next examined the promoter region of the PTEN gene to ascertain whether epigenetic modification, such as hypermethylation, occurred in the CLL cells. The methylation-specific primer (M) and non-methylation primer (UM) were used to amplify the PTEN promoter region from −2476 to −2289 nucleotides upstream of the translation start site, which incorporated a number of CpG sites. We used nested methylation-specific PCR (nMSP) to evaluate the regulatory mechanisms of PTEN expression. The methylation status of the PTEN promoter was evaluated in 75 untreated CLL patients and 25 healthy control cohorts. As shown in Supplemental Figure 4, hypermethylated alleles were detected in only one of 75 (1.33%) CLL samples, but none in the control group. Sequence analysis further confirmed that the CpG islands were hypermethylated. The patient was an old man, peripheral blood examination revealed hyperlymphocyte count. He was asymptomatic, but presented positive ZAP-70 (>20%) and abnormal karyotype of deletion of 11q (site of the ataxia telangiectasia [ATM] gene, clone loss: 274/300). Interestingly, the patient showed a very low PTEN level. It seemed that the level of PTEN was down-regulated in the case at least partly in association with hypermethylation on the promoter region of the PTEN gene. However, the appearance of PTEN gene methylation was relatively rare, which did not represent the majority of the interior in CLL cells.

## DISCUSSION

In CLL, the PI3K pathway is constitutively activated. Activated PI3K is associated with NF-κB activation and expression of BCL-XL and Mcl-1, which mediate inhibition of apoptosis pathway. Additionally, PI3K inhibitor reduces survival signals derived from the BCR or nurse-like cells, and inhibits BCR and chemokine-receptor-induced AKT activation [16]. In particular, miRNA/TP53 feedback circuitry is found to be associated with the pathogenesis of CLL [17]. As is known, p53 and PTEN are involved in sustaining cellular homeostasis and complex regulatory interactions. Namely, cross-talks exist in both p53 and PTEN pathway.

PTEN is a negative regulator of the PI3K/AKT survival pathway, which can promote cell proliferation and contribute to tumorigenesis. Previous studies have shown that PTEN is mutated or inactivated in many types of malignant tumors [7-10]. However, PTEN mutations have only been reported sporadically in leukemia and lymphoma [18-20]. In recent years, several groups identified high frequency of PTEN mutations/deletions in pediatric T-cell acute lymphoblastic leukemia (T-ALL) [21-23]. Jotta and his colleagues found PTEN mutations in primary T-ALL occur almost exclusively in exon 7 [23]. PTEN mutations in exon 7 resulted in predicted C-terminal PTEN protein truncations, which made PTEN protein rapidly degraded [24]. We did not find PTEN exon 5−9 mutations in 154 CLL patients and 200 matched healthy controls except rs555895 PTEN polymorphism in flanking intronic 8 sequences. The rs555895T/T variant was not associated with risk and poor prognosis in CLL. Interestingly, PTEN mutations were also not found in precursor B-cell ALL patients [23]. It appears that there is a world of difference involving PTEN mutations between B-cell and T-cell neoplasms.

DNA methylation is one of the most widespread epigenetic modifications in mammals. Methylation is an important component in cellular processes, including embryonic development, genomic imprinting, X-chromosome inactivation and preservation of chromosome stability [25]. DNA methylation abnormalities have also emerged as one of the most frequent molecular changes in hematological malignancies. Aberrant DNA methylation is the dominant and most well-studied epigenetic alteration in myelodysplastic syndromes (MDS). Various genes, including cell cycle regulators, apoptosis genes, and DNA repair genes, are epigenetically silenced and have roles in pathogenesis and transformation to leukemia [26]. A study from Spain found that PTEN promoter hypermethylation was associated with imatinib resistance in patients with Ph^+^ ALL. They observed reduced PTEN protein was associated with abnormal promoter methylation. Treatment with imatinib and hypomethylating agents increased apoptosis of ALL cells. The use of demethylation drugs might overcome imatinib resistance in Ph^+^ ALL patients [27]. Liu et al found that significantly decreased PTEN protein and low PTEN mRNA levels were partly associated with PTEN methylation in juvenile myelomonocytic leukemia (JMML) [28]. To determine the extent of PTEN hypermethylation and its correlation with PTEN expression in CLL, we first detected the promoter methylation status of PTEN gene in CLL patients. Methylation of PTEN promoter was found in one (1.33%) of the 75 CLL patients, but none in the controls. The patient presented low PTEN expression and some adverse prognostic factors, including elderly age, hyperleukocytosis, positive ZAP-70 and large ATM loss clones. However, the rare incidence rate of PTEN methylation could not represent the total gene expression profile. Therefore, promoter methylation of the PTEN gene is not the principal mechanism of low expression of PTEN in CLL.

As a typical gene regulation pathway, miRNAs interference is another most common epigenetic event in eukaryotic cells. miRNAs interference has been rapidly developed as a tool for functional genomics studies and other applications in mammalians. miRNAs are small non-coding RNA molecule found in plants and animals, which have been discovered to suppress transcription/protein translation of genes by binding to the 3′ UTR of the target genes. By affecting gene expression, miRNAs are likely to be involved in critical biological process, such as cell differentiation, metabolism, apoptosis and hematopoiesis. Abnormal miRNA expression is extensively reported in hematologic neoplasms [29-31], one of the genetic lesions most frequently found in CLL cells involves members of the PTEN/PI3K/AKT signaling pathway. However, few studies have identified miRNAs target PTEN in CLL. Therefore, we examine miRNAs targeting PTEN and evaluate the effect on cell apoptosis. The bioinformatics miRNA databases are used to identify six common conserved PTEN-related miRNAs with high scores. The dual-luciferase assay confirms that the mimics of miR-21, miR-26a and miR-214 significantly suppress the activity of PTEN. The result is consistent with our previous work. Over-expression of miR-21 and miR-26a in CLL patients indicate that miR-21 and/or miR-26a could be the potential therapeutic targets [15].

Subsequently, we examine the effects of antisense oligonucleotides targeted to miR-21, miR-26a and miR-214 in the primary CLL cells. The results show the levels of PTEN mRNA and proteins are up-regulated in transfected cells with miR-26a and miR-214 inhibitors compared with the controls. To investigate transfection efficiency of these three miRNAs inhibitors, we compare changes in these three miRNAs. In spite of numerous studies on miRNAs and PTEN, the different researches got diverse conclusions in PTEN mRNA and protein. Generally, plant miRNAs usually have near-perfect pairing with mRNA targets and induce gene repression through degradation of the target transcripts [32,33]. In contrast, animal miRNAs are able to recognize their target mRNAs by as little as 6-8 nucleotides at the 5′ end of miRNA [34,35]. Therefore, incomplete complementary target mRNA of animal miRNAs usually inhibits gene expression at the level of protein translation [36]. Leone et al [37] reported the levels of PTEN mRNA and protein were up-regulated after transfection of miR-21 inhibitors, and supported partly by some literatures [38,39]. They concluded that transfection of miR-21 inhibitors only resulted in an increase in PTEN protein. Interestingly, Folini and his coworkers [40] found miR-21 was not involved in the regulation of PTEN expression in prostate cancer, which further confirmed that that miRNA exhibited an apparent tissue specific. Moreover, we observe knockdown of PTEN with miR-26a and miR-214 inhibitors induces CLL cell apoptosis. Simultaneously, we found the PTEN mRNA level had an inverse correlation with miR-26a and miR-214 expression in CLL. High expression of miR-26a is associated with advanced clinical Binet stage, p53 aberrations and inferior TTFT, and high expression of miR-214 is only associated with p53 aberrations. Several lines of evidence have shown that miR-26a and miR-214 were overexpressed in cancers and directly targeted PTEN [41-43].

In summary, our study indicates that the down-regulation of PTEN expression in CLL is related to the aberrant expression of miR-26a and miR-214, especially miR-26a, but not PTEN mutations or promoter methylation. Therefore, both miR-26a and miR-214 might play an important role in the pathogenesis of the disease. Restitution of PTEN activity by knockdown of miR-26a and/or miR-214 might provide the potential targets for the CLL intervention.

## MATERIALS AND METHODS

### Patient and clinical samples

One hundred and sixty-seven previously untreated CLL patients were enrolled in the study between February 2008 and September 2013. Another 200 age-matched healthy controls were selected from the local center of Health Examination. This study was approved by the local Internal Review Boards and Ethics Committee. The diagnosis of CLL was based on clinical characteristics, peripheral blood and bone marrow morphology, immunophenotype, and peripheral blood absolute B-lymphocyte count ≥5.0×10^9^/L according to the revised National Cancer Institute (NCI) criteria [44]. Mononuclear cells were isolated from peripheral blood of the CLL patients and healthy donors. Clinical characteristics including gender, age, Binet stage, ZAP-70, CD38, IGHV mutation status, p53 mutation/deletion and cytogenetic abnormalities.

### Cell culture

For preparation of primary cell cultures, CLL cells from 13 untreated patients were isolated from peripheral blood by density gradient centrifugation. The isolated cells were predominantly CLL B cells (>90% CD5^+^CD19^+^) assessed by flow cytometry (Becton Dickinson). Freshly isolated cells were cultured in OPTI-MEM I reduced serum media (Gibgo) supplemented with 10% fetal bovine serum and maintained at 37°C with 5% CO_2_.

### Prediction of miRNA targets and luciferase reporter assay

Bioinformatics miRNA databases were used to identify PTEN-related miRNAs, including miR-Base (http://www.mirbase.org/), target scan human (www.targetscan.org/), miRGen (http://www.diana.pcbi.upenn.edu/cgi-bin/miRGen/v3/Targets.cgi), microRNA.org-Targets and Expression (http://www.microrna.org/microrna/getGeneForm.do). The common conserved miRNAs with higher scores were selected and further validated by dual-luciferase reporter gene assay (Promega). We performed a renilla-luciferase assay with a modified expression pEZX vector containing the complete 3′ UTR region of PTEN cloned in the 3′UTR region of the dual luciferase gene. For dual-luciferase assay, 50 nM, 100 nM, 200 nM and 400 nM of the miR-19a, miR-21, miR-26a, miR-26b, miR-214, miR-1297 mimics and miR-NC were transfected in the 293T cell line together with pEZX vector using lipofectamine 2000. Renilla luciferase activities were measured after 24 h-transfection. The ratios of Renilla versus firefly signals served as a measure of reporter activity normalized for transfection efficiency.

### Transient transfection

The primary isolated CLL cells were plated in 6-well plates at a density of 5.0 ×10^6^ cells/well and transiently transfected with 100 nM of mature miRNA inhibitors of miR-21, miR-214 and miR-26a, and 100 nM of miR-NC (GenePharma) mediated by Lipofectamine 2000 Transfection Reagent (Invitrogen) following the manufacturer's protocol (http://tools.lifetechnologies.com/content/sfs/manuals/Lipofectamine 2000_Reag_protocol.pdf).

### RQ-PCR analysis for mRNA and miRNAs

Whole RNAs were isolated from the treated primary CLL cells using Trizol reagent. RNA and the miRNAs were reversely transcribed into cDNAs by random primers and miRNA-specific reverse primers, respectively. RQ-PCR was performed on the Sequence Detection System ABI Prism (Applied Biosystems) using SYBR Green I gene expression assays for PTEN and β-actin, miRNAs and U6. Those primers for PCR are shown in Supplemental Table 1. Analysis and fold change were determined using the comparative threshold cycle (Ct) method. All samples were run in duplicate. PCR amplification was performed as previously described [14,15].

### PTEN sequencing and mutational analysis

The PTEN gene exons 5−9 and flanking intronic sequences were amplified by PCR. Primer pairs are listed in Supplemental Table 1. Each PCR reaction was performed using 100 ng of genomic DNA, 2×Taq PCR MasterMix 10 μl (Tiangen) and 10 pmol forward and reverse primers in a total volume of 20 μl. Amplification conditions were as follows: exons 5 and 8: 95°C for 10 min followed by 35 cycles at 95°C for 5 s, 57°C for 30 s, and 72°C for 30 s; exons 6, 7 and 9: 95°C for 10 min followed by 35 cycles at 95°C for 5 s, 60°C for 30 s, and 72°C for 30 s; final extension time of 10 min at 72°C. The PCR products were purified and sent to Invitrogen Co. (Shanghai) for sequencing on ABI3730XL 96-capillary DNA Analyzer (Applied Biosystems). Sequences were evaluated using Chromas V2.22 and Mutation Surveyor V4.0.8.

### Apoptosis assay

Detection of apoptotic cells after 24 h-transfection was performed by using Annexin V/PI (Propidium Iodide) flow cytometric assay (Becton, Dickinson and Company). Transfected CLL cells were washed twice with cold PBS and resuspended in Annexin V binding buffer. Cells were incubated in the dark for 15 min at room temperature after addition of 5.0 μl of Annexin V and 5.0 μl of PI. The cells were collected and analyzed by software Cell Quest. The percentage of cells with apoptosis was calculated. Each experiment was conducted in triplicate.

### Western blot analysis

Twenty-four hours after miRNA transfection, transfected CLL cells were lysed in RIPA buffer containing protease inhibitors and protein was extracted. The protein concentration was assayed by BCA (Beyotime) and 25 μg of protein was separated on a 10% SDS-PAGE gel, transferred to PVDF membranes followed by incubating with a murine monoclonal antibody against PTEN (Cell Signaling Technology) at 1:1000 dilutions. The secondary antibody used was rabbit anti-mouse IgG (Cell Signaling Technology) at 1:1000 dilutions at room temperature. The blot was stripped and reprobed with an anti-β-actin antibody (Cell Signaling Technology) to confirm equal loading. Signals were observed with ECL detection reagent (Pierce).

### Methylation analysis by nested methylation-specific PCR

Genomic DNA was extracted from patients's; peripheral blood mononuclear cells by using QIAmp DNA Blood Mini Kit (Qiagen). Bisulfite conversion and subsequent purification of DNA was performed according to EpiTect Bisulfite Kit (Qiagen) protocols. The methylation status of PTEN gene was analyzed by nMSP. The primers were used in the nested, two-step MSP approach. For Step 1, a 20.0 μl reaction volume was used that contained bisulfite-treated DNA template 2.0 μl, 2×Taq PCR MasterMix 10.0 μl (Tiangen), double distilled water (ddH_2_O) 7.0 μl, PTEN flanking forward and reverse primers 0.5 μl (10 μM), respectively. EpiScope Methylated HeLa gDNA (Takara) was used as a positive control in nMSP, which was purified from human HeLa cells and highly methylated using CpG methylase. Reactions were carried out in Thermal Cycler with following cycling parameters: 95°C for 10 min; 35 cycles at 95°C for 5 s, 56°C for 30 s, and 72°C for 30 s; and the last extension was performed at 72°C for 10 min. PCR products from Step 1 reaction were diluted 1:100 in ddH_2_O, 1.0 μl was used as the DNA template in Step 2 of the nMSP. Step 2 reactions were used the following amplification conditions: 95°C for 10 min; followed by 35 cycles at 95°C for 5 s, at 60°C for 30 s, and 72°C for 30 s; and a final extension step at 72°C for 10 min. After nMSP, a 1.5% agarose gel and staining with GoldView (SBS Genetech) was used to identify the products. The final amplified products were sequenced (Invitrogen) and determined the gene of methylated sites.

### Statistical analysis

The protein bands of Western blot were quantified through the Gel-Pro Analyzer 4.0 program for Windows, and the data were normalized β-actin. Mann-Whitney U-test was used when comparing two groups for apoptosis assay, PTEN mRNA and miRNA expression. The genotype and allele frequencies of the polymorphisms were determined by gene counting. Comparisons between categorical variables were examined by the Chi-squared test. All statistical analyses were conducted using the SPSS V17.0 software for Windows and the results were considered to be significant when the P-value was ≤0.05.

## SUPPLEMENTARY MATERIAL AND FIGURES


