# Supplementary material for: miR-26a and miR-214 down-regulate expression of the PTEN gene in chronic lymphocytic leukemia, but not PTEN mutation or promoter methylation
Source: Oncotarget. 2014 Oct 24;6(2):1276–85. doi: 10.18632/oncotarget.2626 (PMC4359232; doi:10.18632/oncotarget.2626)
Supplement: Supplementary file 1 [file oncotarget-06-1276-s001.pdf]

miR-26a and miR-214 down-regulate expression of the PTEN gene in chronic lymphocytic leukemia, but not PTEN mutation or promoter methylation

Supplementary Material

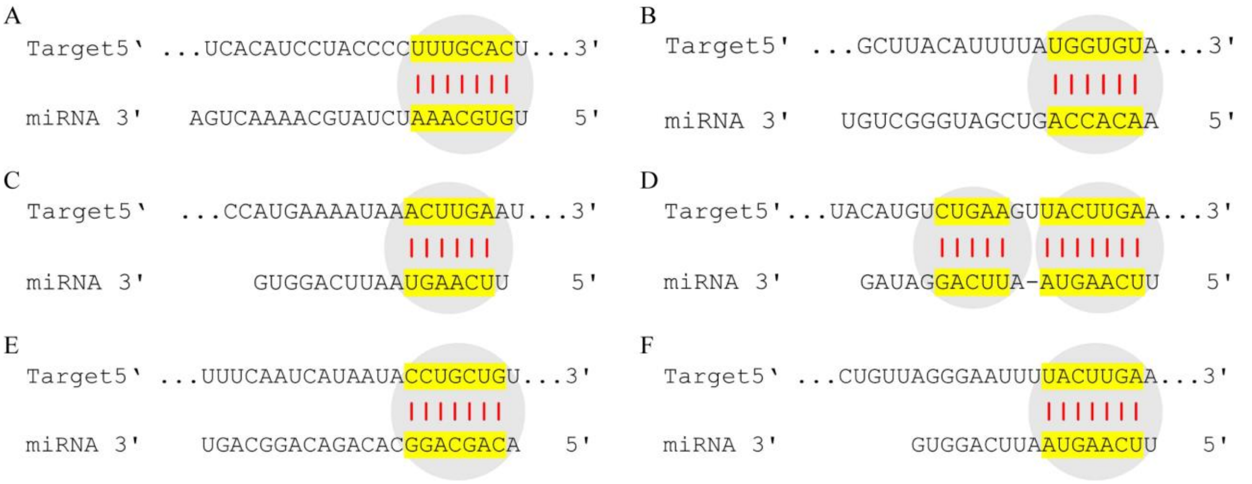

Supplemental Figure 1: The bioinformatics predict the possible binding sites of six miRNAs targeting PTEN. miR-19a (A), miR-21 (B), miR-26a (C), miR-26b (D), miR-214 (E) and miR-1297 (F) selectively bind specific PTEN sequences.

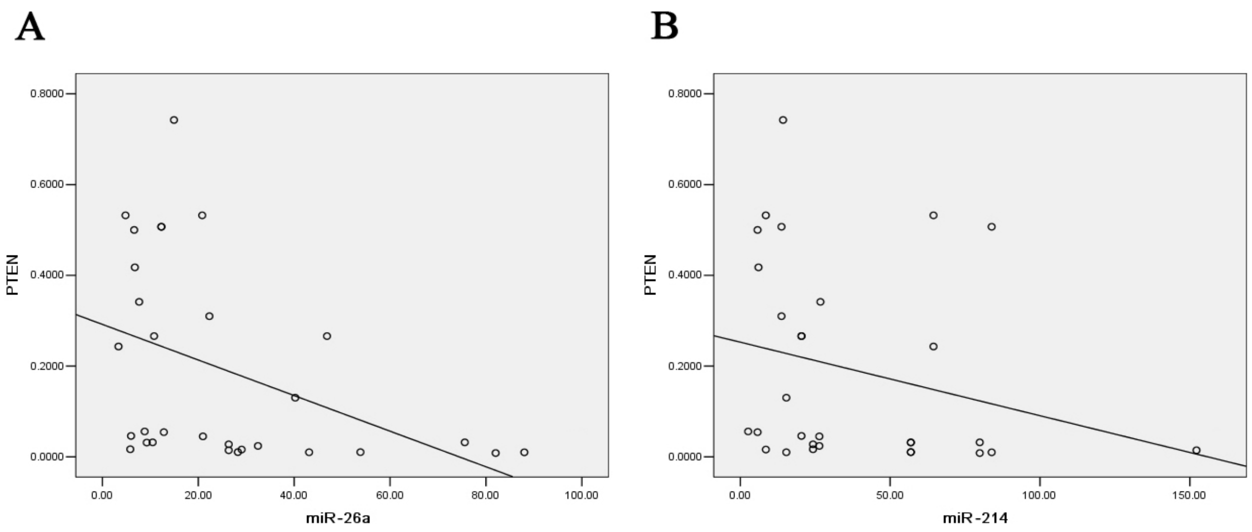

Supplemental Figure 2: The PTEN mRNA level have an inverse correlation with miR-26a (r=-0.563, P=0.001, Supplemental Figure 2A) and miR-214 (r=-0.418, P=0.022, Supplemental Figure 2B) in 30 primary CLL patients (X axis: miR-26a or miR-214 expression, Y axis: PTEN mRNA expression).

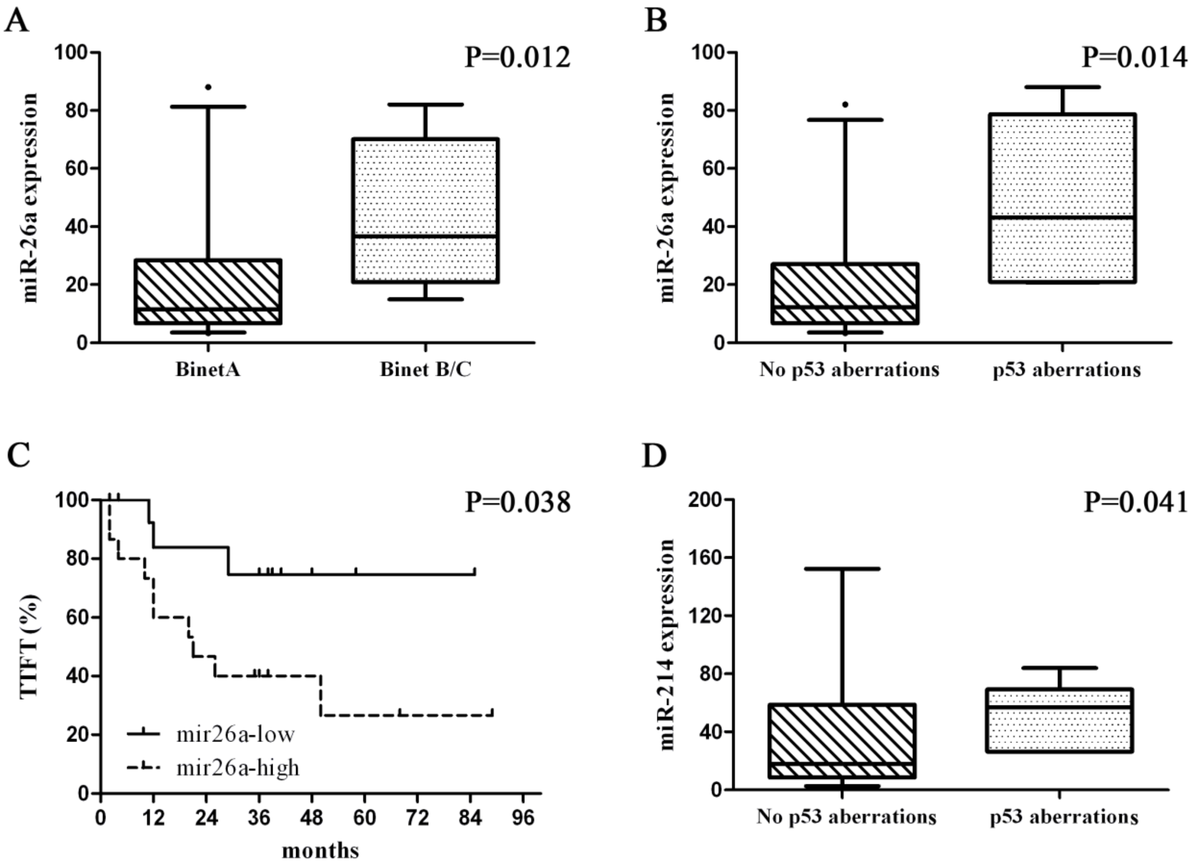

Supplemental Figure 3: High expression of miR-26a correlates with adverse clinical characteristics and inferior time to first treatment (TTFT). High expression of miR-26a was associated with advanced Binet stage (P=0.012, Supplemental Figure 3A), p53 aberrations (P=0.014, Supplemental Figure 3B) and inferior TTFT (P=0.038, Supplemental Figure 3C), but high expression of miR-214 was only associated with p53 aberrations (P=0.041, Supplemental Figure 3D).

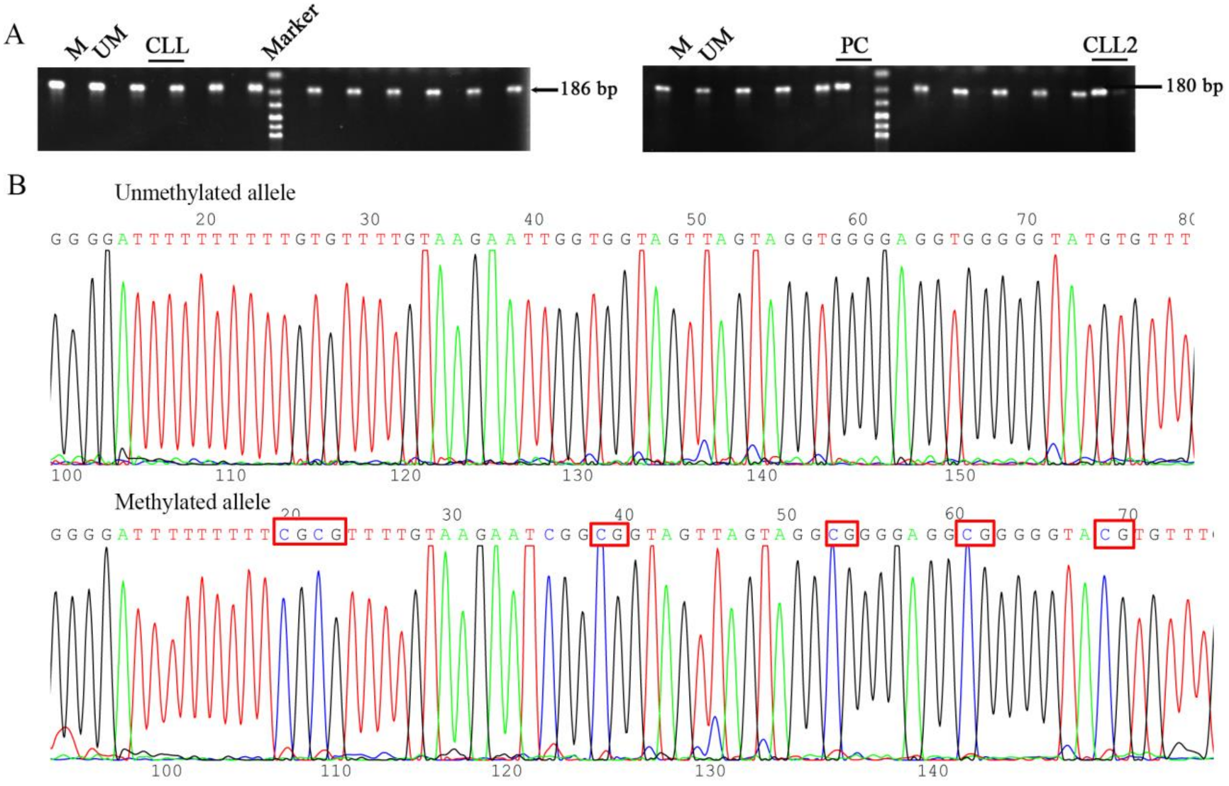

Supplemental Figure 4: PTEN promoter methylation in CLL patients and controls. Methylated alleles are detected in only one of 75 (1.33%) CLL patients. (A) Nested methylation-specific PCR (nMSP) is used to evaluate the methylation status of PTEN promoter. M: amplified products with primer specific for methylated allele (180 bp), UM: amplified products with primer specific for unmethylated allele (186 bp), PC: positive control (Methylated HeLa gDNA from Takara), Marker band: 100-600 bp. (B) Chromatogram of the nMSP products sequence. The bases of the box indicate the methylated CpGs islands (CG) or unmethylated ones (TG).

Supplemental Table 1: Sequences of reverse transcription and PCR primers for miRNA and PTEN

Primer		Sequence (5′-3′)
PTEN-5e	Upstream	GCAACATTTCTAAAGTTACCTAC
	Downstream	TGTTTTCCAATAAATTCTCAGATCCA
PTEN-6e	Upstream	ACGACCCAGTTACCATAGCA
	Downstream	CTCCTGCATAAATTTCAAATGTGGT
PTEN-7e	Upstream	TTGACAGTTTGACAGTTAAAGGCAT
	Downstream	TGTCCTTATTTTGGATATTTCTCCC
PTEN-8e	Upstream	GTTTAACATAGGTGACAGATTTTCT
	Downstream	ACTGCTACGTAAACACTGCT
PTEN-9e	Upstream	AGGCCTCTTAAAGATCATGTTTGT
	Downstream	TCTGACACAATGTCCTATTGCCA
PTEN	Upstream	GCTGTGGTTGCCACAAAGTGCC
	Downstream	GCAGGTAGAAGGCAACTCTGCCA
β-actin	Upstream	AGCGAGCAT CCCCCAAAGTT
	Downstream	GGGCACGAAGGCTCATCATT
U6	Reverse	GTCGTATCCAGTGCAGGGTCCGAGGTATTCGCACTGGATACGACAAAATA
	Upstream	CTCGCTTCGGCAGCACATA
	Downstream	GTGCAGGGTCCGAGGT
miR-21	Reverse	GTCGTATCCAGTGCAGGGTCCGAGGTATTCGCACTGGATACGACTCAACA
	Upstream	GCGGGTAGCTTATCAGACTG
	Downstream	GTGCAGGGTCCGAGGT
miR-26a	Reverse	GTCGTATCCAGTGCAGGGTCCGAGGTATTCGCACTGGATACGACAGCCTA
	Upstream	GCCTTCAAGTAATCCAGGA
	Downstream	GTGCAGGGTCCGAGGT
miR-214	Reverse	GTCGTATCCAGTGCAGGGTCCGAGGTATTCGCACTGGATACGACACTGCC
	Upstream	GCACAGCAGGCACAGACAG
	Downstream	GTGCAGGGTCCGAGGT
